# Effect of Vestibular Rehabilitation on Spontaneous Brain Activity in Patients With Vestibular Migraine: A Resting-State Functional Magnetic Resonance Imaging Study

**DOI:** 10.3389/fnhum.2020.00227

**Published:** 2020-06-12

**Authors:** Li Liu, Xiaofei Hu, Yixin Zhang, Qi Pan, Qunling Zhan, Ge Tan, Kuiyun Wang, Jiying Zhou

**Affiliations:** ^1^Department of Neurology, The First Affiliated Hospital of Chongqing Medical University, Chongqing, China; ^2^Department of Neurology, Chongqing Renji Hospital, University of Chinese Academy of Sciences, Chongqing, China; ^3^Department of Radiology, Southwest Hospital, Third Military Medical University (Army Medical University), Chongqing, China; ^4^Department of Neurology, The Jintang First People’s Hospital, Sichuan University, Sichuan, China

**Keywords:** functional magnetic resonance imaging, vestibular rehabilitation, vestibular migraine, resting state, amplitude of low-frequency fluctuation

## Abstract

Previous studies have shown that vestibular migraine (VM) is a cerebral disease with recurrent vertigo. Vestibular rehabilitation (VR) is an effective type of physical therapy for minimizing vestibular symptoms, as it improves vestibular compensation in patients with VM. Currently, the cerebral regions that are associated with the pathogenesis of VM are largely unknown. To further understand the underlying mechanisms of VM, we performed resting-state functional magnetic resonance imaging (fMRI) before and after 1 month of VR in 14 patients with VM. The Dizziness Handicap Inventory (DHI), the 36-Item Short-Form Health Survey (SF-36), the Hamilton Depression Scale (HAMD) and the Hamilton Anxiety Scale (HAMA) scores were included as clinical outcomes. The amplitude of low-frequency fluctuation (ALFF) was assessed to characterize spontaneous brain activity. The correlations between the clinical characteristics and ALFF values were assessed. After 1 month of VR training, the DHI scores in patients with VM were significantly lower than those at baseline (*p* = 0.03), as were the HAMA scores (*p* = 0.02). We also found that the ALFF values in the left posterior cerebellum of VM patients increased significantly after 1 month of VR training. Moreover, the ALFF values in the left cerebellum were inversely correlated with the patients’ DHI scores. Overall, this study showed that VR exercise for 1 month has a positive effect on vestibular symptoms in patients with VM. Asymmetric cerebellar hyperactivity might be a functional compensation for vestibular dysfunction in patients with VM.

## Introduction

Vestibular migraine (VM) is a subtype of migraine that commonly causes paroxysmal vertigo, and its prevalence has been reported to be as high as 1% in the general population (von Brevern et al., 2007). The correlation between migraine and vestibular symptoms is not accidental; as many as 50% of patients with migraine experience dizziness or vertigo (Vukovic et al., 2007). The Barany Society and the International Headache Association have recently classified VM as a distinct entity: vestibular symptoms are viewed as symptoms associated with migraines. In our previous study, we showed that VM was present in 12.2% of patients with dizziness or vertigo in our neurological outpatient clinic, and vertigo attacks appeared, on average, 6 years after the onset of migraine in patients with VM (Zhang et al., 2016). Vertigo significantly increases the risk of falls (Agrawal et al., 2009), and patients often avoid rapid movements and social activities. When vestibular symptoms recur frequently, patients are more inclined to develop psychological problems, such as anxiety and depression (Yardley, 2000), which seriously affect the quality of life (Jeong et al., 2010).

The treatment options of VM are primarily based on those of migraine (Obermann and Strupp, 2014). Studies have shown that physical methods, such as vestibular rehabilitation (VR), are effective in improving vestibular symptoms among patients with VM (Whitney et al., 2000; Vitkovic et al., 2013). VR is a physical therapy that aims to improve patients’ functional recovery and quality of life-based on the learned strategies (Lacour and Bernard-Demanze, 2015). Vestibular compensation is a complex neurochemical reaction process, and the brain regions that are responsible for the genesis of VM are unclear.

Resting-state functional magnetic resonance imaging (fMRI) is an important method to study spontaneous brain function at rest. Low-frequency fluctuations in blood oxygen level-dependent (BOLD) fMRI signals are considered to be physiologically meaningful in assessing spontaneous neural activity in the brain (Goncalves et al., 2006; Shmuel and Leopold, 2008). Migraine is a cerebral disease (Russo et al., 2014; Wang et al., 2016); however, to the best of our knowledge, fMRI studies have not been performed in patients with VM after VR. Therefore, we designed a prospective study to observe the amplitude of low-frequency fluctuation (ALFF) changes in brain regions after vestibular compensation through VR, which might yield meaningful insights into this disease and reveal possible mechanisms by which VR improves patients’ symptoms. Moreover, we also investigated the relationship between clinical indicators and ALFF values before and after VR to identify potential influencing factors.

## Materials and Methods

### Subjects

From October 2017 to October 2018, 19 right-handed VM patients were recruited from the Department of Neurology of the First Affiliated Hospital of Chongqing Medical University and Chongqing Renji Hospital at the University of Chinese Academy of Sciences. VM is mainly diagnosed based on the patient’s clinical history. Vertigo or dizziness can occur before a headache, as well as during or after a headache (Cutrer and Baloh, 1992; Neuhauser et al., 2001). The main complaints of our patients were dizziness or vertigo, and all of these patients were first diagnosed with VM and had not taken any mediations for migraine prophylaxis. All subjects completed 1 month of VR training and functional magnetic resonance examinations before and after the treatment. The Ethical Committee of Chongqing Medical University approved this study, and all patients provided informed consent for their participation in this study. Five patients were excluded for the following reasons: one patient was pregnant, and four failed to complete the VR training.

All patients met the International Classification of Headache Disorders III criteria of VM and underwent vital sign assessments, including neurological examinations. No patients had a history of stroke or severe neurological activity disorder. A standardized questionnaire was administered to record the patients’ clinical characteristics and demographic information. VR was provided by two therapists who assessed the subjects and devised a video to guide the home exercise program. The therapists reviewed the progress of the patients and then added additional exercises, depending on each individual’s improvements or difficulties. All participants completed a home exercise diary to record their exercise plans and compliance.

Brain MRI scans (T1- and T2-weighted images) were inspected by an experienced neuroradiologist. Patients did not have any vertigo episodes for at least 24 h before the scan. If the patients had an episode within this window, they were rescheduled.

The exclusion criteria were as follows: (1) participation in VR in the previous 6 months; (2) a history of cerebrovascular disease; (3) pre-existing neurological or psychiatric disorders (including a history of seizures, cognitive impairment, uncorrected visual impairment, bilateral vestibular disease, previous vestibular neuritis, secondary somatic dizziness, severe depression or claustrophobia); (4) the use of an electrically sensitive biomedical device (e.g., cardiac pacemakers or cochlear implants); (5) metal clips in the brain; and (6) pneumonia at the time of enrolment.

All VM patients were allowed to continue with prophylactic and acute pain medications as usual (Vitkovic et al., 2013). Throughout this 4-week study period, no new medications or physical therapy treatments were started.

### Clinical Assessment

The Dizziness Handicap Inventory (DHI) was used for the vestibular dysfunction assessment. Quality of life was measured by the 36-Item Short-Form Health Survey (SF-36). The HAMD and HAMA were administered.

The DHI is a reliable and effective self-report indicator for patients with vertigo. The higher the DHI score is, the higher the risk of falls in patients with balance and vestibular dysfunction.

The 36-Item Short-Form Health Survey (SF-36) is a generic multidimensional measure of health-related quality of life (Jenkinson et al., 1993). It comprehensively summarizes the individual’s quality of life from eight aspects: (1) physical functioning; (2) social functioning; (3) role limitations due to physical health (role-physical); (4) role limitations due to emotional problems (role-emotional); (5) mental health; (6) vitality; (7) bodily pain; and (8) general health.

The total score of the Hamilton Depression Scale (HAMD) reflects the severity of depressive symptoms (Hamilton, 1960). The total score of the Hamilton Anxiety Scale (HAMA) indicates the severity of anxiety symptoms (Hamilton, 1959). A low total score indicates mild depressive or anxiety symptoms, while a high score indicates severe symptoms.

### Vestibular Rehabilitation Methods

The patients were asked to relax before VR training by slowing the breath to 4–6 s for each inhale and exhale and performing a training protocol for 10 min twice a day for 4 weeks. The training protocol as follows:

(1) Move your head from side to side for 10 s and look in the direction your head is rotated toward. Repeat this motion once every 10 s.
(2) Nod for 10 s. Look in the direction your head is rotated toward. Repeat this motion once every 10 s.
(3) While holding a small object that is static and located approximately 30 cm in front of you, move your head back and forth while maintaining your gaze on the object.
(4) Hold the object still at approximately 30 cm in front of you. While nodding, maintain your line of sight on the object.
(5) To gradually increase the difficulty, move from sitting to standing, from standing to walking, and from a hard surface to an uneven surface.

### Magnetic Resonance Imaging Data Acquisition

fMRI images were collected using a Siemens Tim Trio whole-body MRI system, and the field intensity was 3.0 T. No subjects fell asleep during the procedure. To acquire resting-state functional images, conventional localizer scans, and T2 anatomic scans were performed, and the following parameters were used for the echo-planar imaging (EPI) sequences. There were 36 axial slices, and the thickness of each slice was set to 4 mm, with no slice gap. In addition, other setting parameters are as follows: repetition time = 2,000 ms, echo time = 30 ms, flip angle = 90°, field of view = 192 mm × 192 mm, and data matrix size = 64 × 64. Therefore, the voxel size was 3 mm × 3 mm × 3 mm, and a total of 240 volumes were acquired.

### Data Processing

SPM8 software^1^ was used. The first 10 volumes were discarded to ensure that the participants adapted to the scanning environment and to allow for scanner calibration. Then, the EPI imagines were corrected for the slice timing and realigned according to the head motion (subject data with head motion exceeding 1.5 mm in any direction or more than 1° of rotation were excluded; Zhao et al., 2014; Gao et al., 2016; Wang et al., 2016). Afterward, all data were spatially normalized into the Montreal Neurological Institute EPI space, and each voxel was resampled isotropically to 3 mm × 3 mm × 3 mm. Finally, an isotropic Gaussian kernel with full-width at half-maximum = 4 mm was used to smooth the resting state images spatially.

The ALFF values reflect the activity in the local brain regions (Zang et al., 2007) and were analyzed using open-source software REST (v.1.8^2^). Regarding the preprocessing steps, to remove the linear trend in the time series and reduce the effect of low-frequency drifting and high-frequency physiological noise, the data were bandpass filtered (0.01–0.08 Hz). Then, the power spectrum was identified using a fast Fourier transform, which transformed the time series to a frequency domain. Next, the square root of the power spectrum was calculated and averaged across 0.01–0.08 Hz at each voxel. This averaged square root was taken as the ALFF, which was assumed to reflect the absolute intensity of spontaneous brain activity (Wang et al., 2012).

As micromovements across volumes might affect functional connectivity at rest, as suggested in a prior study (Yan et al., 2013), framewise displacement (FD) values were calculated for each subject to identify the temporal derivatives of the movement parameters. One subject who had FD > 0.5 mm on more than 35 volumes was excluded from the group-level analyses. In the group statistical analyses of ALFF, the mean FD was included as a covariate.

### Statistical Methods

The DHI, HAMD, HAMA, and SF36 variables before and after VR were compared using paired samples *t*-test and SPSS 18.0 (SPSS Inc., Chicago, IL, USA). Differences between groups with *P* < 0.05 were considered significant. To examine the differences in the ALFF between the baseline and after VR, a paired *t*-test of the ALFF maps was performed by REST v.1.8 software, with age and gender as covariates. Multiple comparison corrections were performed using a Gaussian random field (GRF) at *p* < 0.001 and *z* > 2.65. Finally, we also performed correlation analyses between the ALFF values and clinical characteristics, including changes in the DHI scores. Because gender had no significant effect on the results of the correlation analyses, partial correlation analysis was performed using SPSS 18.0 with age as a covariate. The effective threshold was set at *p* < 0.05.

## Results

### Clinical Characteristics of the Patients

During the study period, 14 patients were newly diagnosed with VM (nine females/five males, 43.86 ± 11.61 years, range 25–60 years). The average age of the female subjects was 42.22 ± 12.82 years, and the average age of male subjects was 46.80 ± 9.63 years. Table 1 shows that the mean ages at the onset of migraine and vertigo were 20.21 ± 8.11 and 22.14 ± 10.08 years, respectively. Vertigo attacks always associated with migraine were reported in eight patients (57%). The most common type of vertigo was spontaneous vertigo (86%), followed by head motion-induced vertigo (29%). The frequency of vertigo attacks was between 1 and 6 days per month (2.86 ± 2.18 days/month). Most vertigo episodes lasted from 5 min to 24 h. Six (43%) patients had vertigo episodes lasting 5–60 min. Five (36%) patients had vertigo attacks lasting from 1 to 24 h. A family history of migraine was reported in eight (57%) patients, and vertigo in first-degree relatives was reported in four (29%) patients.

**Table 1:** Clinical characteristics of patients with vestibular migraine (VM).

Characteristics	Mean ± SD
Gender (male/female)	5/9
Age (years)	43.86 ± 11.61
Male	46.80 ± 9.63
Female	42.22 ± 12.82
Age at onset	
Migraine	20.21 ± 8.11
Vertigo attacks	22.14 ± 10.08
Headache	
Always	8/14 (57%)
Sometimes	6/14 (43%)
Vertigo history (years)	21.86 ± 14.11
Frequency of vertigo attacks (days/month)	2.86 ± 2.18
Type of vertigo, *N* (%)	
Spontaneous	12/14 (86%)
External	9/14 (64%)
Internal positional	3/14 (21%)
Visually induced	2/14 (14%)
Head motion-induced	4/14 (29%)
Duration of vertigo attacks, *N* (%)	
<5 min	3/14 (21%)
5–60 min	6/14 (43%)
1–24 h	5/14 (36%)
24–72 h	2/14 (14%)
>72 h	2/14 (14%)
Family history (1st-degree relatives), *N* (%)	
Migraine	8/14 (57%)
Chronic vertigo	4/14 (29%)

### Comparison of the Clinical Assessment Before and After VR

Table 2 summarizes the clinical assessment results of the subjects at baseline and after 1 month of VR, including the DHI, HAMA, and HAMD scores. The eight domains of the SF36 scores were analyzed.

**Table 2:** The clinical assessment of VM patients before and after vestibular rehabilitation (VR).

Variables	VM patients before VR (*N* = 14) Mean ± SD	VM patients after VR (*N* = 14) Mean ± SD	*p*-value
Dizziness Handicap Inventory	33.71 ± 18.45	23.14 ± 16.03	0.003^a^
Hamilton Anxiety Scale	10.43 ± 5.65	6.00 ± 3.80	0.002^a^
Hamilton Depression Scale	9.29 ± 6.34	6.43 ± 5.27	0.064
SF36 domains			
Physical functioning	88.93 ± 12.28	96.07 ± 2.89	0.062
Social functioning	92.86 ± 17.48	96.43 ± 7.64	0.414
Role-physical	55.36 ± 35.36	82.14 ± 33.15	0.037^a^
Role-emotional	57.14 ± 51.36	85.71 ± 31.25	0.047^a^
Mental health	58.57 ± 20.73	64.57 ± 15.52	0.246
Vitality	63.93 ± 15.34	71.07 ± 14.17	0.222
Bodily pain	75.00 ± 26.83	83.29 ± 16.60	0.340
General health	53.07 ± 31.51	63.21 ± 17.01	0.198

*^a^Results from paired *t*-test of the comparison in VM patients before and after treatment, *p*-value < 0.05*.

The results showed that the DHI scores of the patients after completing VR training were significantly lower than those at baseline (*p* = 0.03), and the HAMA scores also significantly decreased (*p* = 0.02), but there was no significant difference in the HAMD scores before and after 1 month of VR training.

The SF36 results showed that the role-physical (*p* = 0.037) and role-emotional (*p* = 0.047) scores were significantly higher in patients after VR than at baseline. There were no significant differences in the aspects of physical functioning, social functioning, mental health, vitality, bodily pain, or general health.

### Resting-State ALFF Values Before and After VR

**Figure 1:**
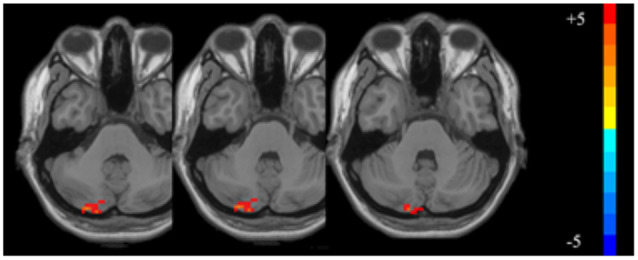
The significantly altered amplitude of low frequency fluctuation (ALFF) map of the left posterior cerebellum in vestibular migraine (VM) patients after vestibular rehabilitation (VR; GRF corrected: *p* < 0.001, *z* > 2.65). The color bar denotes the *t*-value.

**Figure 2:**
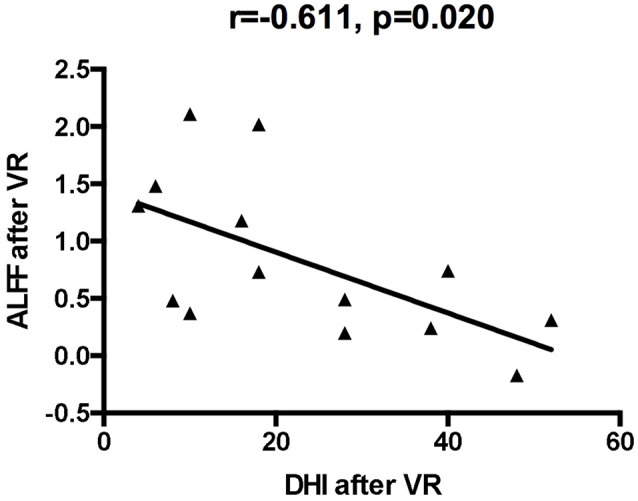
The correlation of ALFF values of the left cerebellum posterior lobe with dizziness handicap inventory (DHI) values after VR.

Compared with the baseline values, the ALFF values after VR were significantly higher in the left posterior cerebellum among patients with VM (GRF corrected: *p* < 0.001, *z* > 2.65; Table 3, Figure 1). Also, the secondary analyses revealed a negative correlation between the ALFF values in the left posterior cerebellum and the DHI scores in the patients with VM (*r* = −0.611, *p* < 0.05, uncorrected; Figure 2). No significant correlations were found between other clinical features of the VM patients and the activity in the left posterior cerebellum.

**Table 3:** Brain regions with significant amplitude of low frequency fluctuation (ALFF) differences between VM patients before and after VR.

Region	MNI coordinates			Peak T-value	Cluster size (mm^3^)
	*x*	*y*	*z*		
L.Cerebellum Posterior Lobe	27	87	36	4.420	37

*MNI, Montreal Neurological Institute; L, left; (GRF corrected: *p* < 0.001, *z* > 2.65)*.

## Discussion

This study demonstrated the clinical benefits of VR in improving vestibular symptoms, mood disorders, and quality of life. More importantly, this is the first clinical study based on fMRI to explore the potential mechanisms which help explain the improvement of the symptoms of patients with VM by VR.

This study presented the clinical features of vestibular migraine. The female preponderance was confirmed in this study. The onset age of vertigo in patients with VM was consistent with those reported in previous studies (Neuhauser et al., 2001; Radtke et al., 2011). The most common vertigo type was spontaneous vertigo, and the incidence of external vertigo was higher than that of internal vertigo. The duration of vertigo attacks was less than 72 h in 86% of patients. All patients were clinically examined, and their DHI scores were used to assess their degree of dizziness. Correlation analyses were performed between the ALFF values and clinical data. Interestingly, the correlation analyses showed that the ALFF values in the left cerebellum were inversely correlated with the patient’s DHI scores after VR, indicating a positive correlation between the clinical outcomes and the adaptability of patients’ vestibular system.

In our study, the total DHI score significantly decreased after VR, suggesting that 1 month of VR training can effectively alleviate vestibular symptoms in patients with VM, which is consistent with the results in previous studies (Wrisley et al., 2002; Gottshall et al., 2005; Sugaya et al., 2017). After VR training, most patients reported mild to moderate relief of vertigo or dizziness, and some patients had significant or even complete relief of symptoms. This effect was not associated with the patient’s age or sex or duration of the disease (Whitney et al., 2002; Cohen and Kimball, 2003). Boldingh studied VM and migraine patients with vestibular dysfunction during the interictal period (Boldingh et al., 2013). VM with vestibular dysfunction was twice as common as migraine, and the VM patients were worse than migraine patients in maintaining balance. VR with gaze stabilization exercises improved the gain of the vestibular ocular reflex, improving visual acuity during head movements and reducing the symptoms of dizziness and vertigo (Meldrum and Jahn, 2019). The occipital lobes, midbrain, and cerebellum played important roles in recalibrating the vestibulo-ocular reflex (VOR) gain (Sulway and Whitney, 2019). The results showed that the value of vestibular rehabilitation for VM patients is self-evident, and the cerebellum plays a role in the treatment.

VM patients showed significantly lower HAMA scores after VR than at baseline, but there was no significant difference in the HAMD scores. The cerebellum plays an important role in cognitive, sensorimotor, and emotional information processing (Hu et al., 2008). VM patients have a high risk of psychiatric comorbidities, particularly anxiety and depressive disorders (Lahmann et al., 2014). Migraineurs have exhibited hyperactivation in the cerebellum while viewing negative stimuli (Wang et al., 2017). Besides, emotion-related activation has also been reported in the cerebellum of healthy subjects (Moulton et al., 2011), suggesting that specific regions of the cerebellum may be involved in the encoding of generalized aversive processing. The present study also showed that after active VR training, the anxiety status of patients with VM improved. However, previous studies have shown controversial results on emotional scores in patients with migraines after VR (Whitney et al., 2000; Sugaya et al., 2017). Therefore, more prospective studies on the relationship between the VR training period and emotional changes should be conducted.

VR is an effective treatment for patients with benign paroxysmal positional vertigo and Meniere’s disease, as it markedly improved patients’ quality of life (Socher et al., 2012). However, it has never been evaluated among patients with VM. We administered the SF36 to evaluate the quality of life. The results showed that patients’ quality of life significantly improved. After VR, there were significant improvements in the role-physical and role-emotional SF36 domains. The primary goals of VR therapy are to improve individuals’ dynamic performance through the learned strategies, yield improved functional recovery, and improve patients’ quality of life (Lacour and Bernard-Demanze, 2015). Emotional improvements have also been shown to be related to the positive effects of VR therapy (Meli et al., 2007). However, the mechanisms of VR are still unclear. Our research showed that cerebellar function might be involved. The cerebellum can improve vestibular function through a vestibular compensation mechanism, which might also participate in the modulation of emotional processing (Minichino et al., 2014).

Recent neuroimaging studies have shown that patients with VM exhibit extensive brain dysfunction, which involves cognitive behavior and sensory-motor integration secondary to vestibular, visual, proprioceptive, and somatosensory afferents (Russo et al., 2014; Shin et al., 2014). Subclinical vestibular-cerebellar dysfunction has been found in patients with migraine, according to the results of various vestibular and cerebellar tests (Harno et al., 2003; Qin et al., 2019). ALFF changes in the posterior bilateral cerebellar lobe have been found in migraine patients (Wang et al., 2016). Both a PET study during VM episodes (Shin et al., 2014) and an fMRI study during caloric vestibular stimulation in patients with VM (Russo et al., 2014) showed increased cerebellar metabolism. However, these studies were cross-sectional and did not involve therapeutic interventions. In this study, VM patients underwent VR for 1 month. Compared with baseline, significant hypermetabolism of the cerebellum was observed, suggesting the cerebellum plays an important role in VR training. This role may associate with the adaptive mechanism used to achieve vestibular compensation.

VR can be used to improve vestibular compensation, and vestibular compensation includes static and dynamic processes (Lacour et al., 2016). Adaptation, including sensory and behavioral substitutions, is the main mechanism of dynamic functional recovery (Lacour and Bernard-Demanze, 2015). Sensory substitution plays an important role in individuals’ ability to compensate for the vestibular loss (Lacour and Bernard-Demanze, 2015). Vision and proprioception are the main sensory substitution sources in the recovery of patients’ dynamic function. Previous fMRI studies provided evidence for sensory substitution in patients with vestibular neuritis. A significant increase in gray matter volume was observed in the vestibular cortex, bilateral hippocampus, visual cortices, and cerebellum at 3 months after the unilateral loss of vestibular dysfunction. In particular, the increases in gray matter volume in the visual cortices and cerebellum were found to be associated with the recovery of balance (Hong et al., 2014). At 6 months after a vestibular neuritis attack, the BOLD signal in the primary visual cortex changes (Roberts et al., 2018). Behavioral substitution has been shown to promote the learning of new strategies through active head movements, conceal inadequate VOR responses, and restore gaze stability quickly (Tian et al., 2002). Functional MRI after vestibular stimulation in healthy subjects showed that the posterior cerebellum is significantly activated during the leftward movement of the eyeball (Roberts et al., 2017). This result indicated that the cerebellum might play an important role in motor learning. Our study showed increased ALFF values in the posterior cerebellum of the left side after treatment compared with before treatment. Thus, our results suggest enhanced spontaneous activity of the cerebellum in VM patients after one-month VR training.

Our study showed that there was an asymmetry in cerebellar activation, which presented as significantly increased ALFF values in the posterior lobe of the left cerebellum. Neuroimaging has previously demonstrated asymmetry in the cerebellar function (Solodkin et al., 2001; Xiang et al., 2003). One study revealed a predominant activation in the left cerebellar hemisphere during optokinetic nystagmus (Ruehl et al., 2017). VR improves VOR through oculomotor and gaze stability exercises (Morimoto et al., 2011), improves visual acuity during head movement, and reduces the symptoms of dizziness and vertigo (Meldrum and Jahn, 2019). This dynamic process may rely on the cerebellum to recalibrate the VOR gain. We also found that a complementary left cerebellar was predominant for VR. Existing literature showed that the right hemisphere was predominant for visuospatial and vestibular processing in humans (Dieterich et al., 2003). Considering that most of the cortical cerebellum is projected contralaterally, the mechanism supporting VR to improve vestibular symptoms is mainly compensated by the left cerebellum. In this study, the increased spontaneous activity of the cerebellum indicates that the cerebellum, especially the left posterior cerebellum, plays an active role in vestibular compensation. Asymmetric cerebellar hyperactivity might be a functional compensation in patients with VM.

## Data Availability Statement

The raw data supporting the conclusions of this article will be made available by the authors, without undue reservation, to any qualified researcher.

## Ethics Statement

The studies involving human participants were reviewed and approved by the Ethical Committee of the Chongqing Medical University. The patients/participants provided their written informed consent to participate in this study.

## Author Contributions

JZ and KW designed the study and edited the manuscript. LL recruited participants, performed the statistical analysis, and wrote the manuscript. XH collected the MRI data and performed the statistical analysis. YZ, QP, QZ, and GT recruited participants and collected the clinical data. All authors read and approved the final manuscript.

## Conflict of Interest

The authors declare that the research was conducted in the absence of any commercial or financial relationships that could be construed as a potential conflict of interest.

## Footnotes

^1^http://www.fil.ion.ucl.ac.uk/spm/

^2^http://resting-fmri.sourceforge.net

## Abbreviations

VM: vestibular migraine
VR: vestibular rehabilitation
ALFF: amplitude of low-frequency fluctuations
DHI: Dizziness Handicap Inventory
fMRI: functional magnetic resonance imaging
BOLD: blood oxygen level-dependent
SF-36: the 36-Item Short-Form Health Survey
HAMD: Hamilton Depression Scale
HAMA: Hamilton Anxiety Scale
EPI: echo-planar imaging
GRF: Gaussian random field
FD: framewise displacement
VOR: vestibulo-ocular reflex.
